# Mapping the susceptibility of persons with disabilities to landslides in a highland landscape of Bushika Sub County, Mount Elgon, Eastern Uganda

**DOI:** 10.4102/jamba.v14i1.1266

**Published:** 2022-05-25

**Authors:** Martin Ssennoga, Frank Mugagga, Daniel L. Nadhomi, Yeeko Kisira

**Affiliations:** 1 Department of Disability Inclusive Disaster Risk Reduction, National Union of Disabled Persons of Uganda, Kampala, Uganda; 2 Department of Geography, Geo-informatics and Climatic Sciences, School of Forestry, Environment and Geographical Sciences, College of Agriculture and Environmental Sciences, Makerere University, Kampala, Uganda; 3 Department of Geography and Social Studies, Faculty of Arts and Social Sciences, Kyambogo University, Kampala, Uganda

**Keywords:** susceptibility, persons with disabilities, landslide risk, inclusive disaster programmes, Highland landscape, Mount Elgon

## Abstract

Terrain parameters such as slope aspect, angle, curvature, stream power and altitude have been noted to spur landslide occurrence as well as, acting as a hindrance to evacuation efforts. Yet, persons with disabilities (PWDs) are seldom given priority during rescue and recovery programmes during pre- and post-disaster evacuation. The study was guided by two objectives, namely, (1) to map the landslide risk for households of PWDs and (2) to investigate the disability type that is perceived to be most affected by landslides. A cross-sectional household survey was adopted employing snowball sampling, Key Informant Interviews (KII), and Focus Group Discussions (FGDs) for primary data collection. A 30-m Shuttle Radar Topography Mission (SRTM) Digital Elevation Model (DEM) was used for terrain spatial landslide risk analysis in ArcGis 10.8 and System for Automated Geoscientific Analyses (SAGA) tools. A one-sample *t*-test in Statistical Package for Social Sciences (SPSS) version 23 was used to analyse the score values on a five-point Likert scale to ascertain the perceived landslide effect on the different disability categories. Qualitative data was subjected to content analysis. We found out that majority of PWDs live in high-risk landslide zones with 1400 m – 1700 m, S-E, 10–80, > 10, and –0.8–0.13 of altitude, aspect, slope angle, Stream Power Index (SPI), and slope curvature, respectively. T-test results revealed that blind and deaf-blind were perceived as most affected by landslides with *t*(31) = 58.42, mean = 4.7, *p* < 0.0001, and *t*(31) = 34.8, mean 4.6, *p* < 0.0001. The deaf people were perceived to also be highly affected by landslides with *t*(31) = 34.4, mean = 3.9, *p* < 0.0001. In conclusion, PWDs in Bushika were highly susceptible to landslide hazards and yet considered as a minority for rescue and recovery during landslide occurrences. We recommend for prioritisation of inclusive disaster programmes such as disaster training, relocation, and resettlement to reduce vulnerability and enhance landslides disaster resilience of PWDs especially in high-risk areas.

## Introduction

Persons with Disabilities (PWDs) are regarded as one of the minorities and marginalised groups and highly vulnerable to disasters, especially landslides (Global Facility for Disaster Reduction and Recovery [GRDRR] 2017). People with disabilities represent about 12% of the world’s population (Bongo, Dziruni & Muzenda-Mudavanhu 2018). *The American Disability Act* (1990) defines an individual with a disability as a person who has a physical or mental impairment that substantially limits one or more major life activities, a person who has a history or record of such an impairment, or a person who is perceived by others as having such an impairment (United States Department of Justice [USDJ] 2020). Also, United Nations Convention on Rights of Persons with Disabilities (UNCRPD) (2010) describes PWDs as those who have long-term physical, mental, intellectual, or sensory impairments which in interaction with various barriers may hinder their full and effective participation in society on an equal basis with others (Rohwerder 2015; UN 2010). Also, disabilities include those with difficulty in the body functions, for example, difficulty in hearing, difficulty in speaking and conveying messages, difficulty in moving around and using other body parts, difficulty in seeing, strange behaviour, epilepsy, difficulty in learning, Leprosy as also listed (NICHCY 2012). The Uganda National Policy on Disability 2006, describes PWDs as people with permanent and substantial functional limitation of daily life activities caused by physical, mental or sensory impairment and environmental barriers resulting in limited participations (Ministry of Gender, Labour and Social Development [MGLSD] 2006). It includes all people with difficulty in seeing, hearing, speaking and conveying messages, difficulty moving around and using other body parts, epilepsy, strange behaviour, leprosy, difficulty in learning, loss of feeling and multiple or a combination of two or more other disabilities.

Globally, PWDs normally live in isolated locations due to stigma and discrimination attached to disability (WHO 2011). This makes it harder to reach them with support during and after the hazard (Gomathy 2015; WID 2018). The key elements at risk are the homes and lives of PWDs. The Sendai Framework for Disaster Risk Reduction (SFDRR) of 2015 emphasises full participation of PWDs in the community programmes to enhance their resilience towards disasters (United Nations International Strategy for Disaster Reduction [UNISDR] 2015). This is because, there is a strong association between disability and disaster impact in most communities (Gutnik & Roth 2018; Hosseinpoor et al. 2013). Monitoring the risk exposure of PWDs to climate-induced landslide disasters remains one of the most critical concerns because of the high and disproportionate risk PWDs encounter in the face of risks and humanitarian emergencies.

In Uganda, the total number of PWDs is estimated to be more than 4.4 million with Bududa district having a higher disability prevalence averaged at 15.2% (Uganda Bureau of Statistics [UBOS] 2019). However, the district lies in the Mount Elgon region, a hotspot for steep slopes and a series of landslide hazards (Namono et al. 2019). The estimated number of deaths due to landslides in half a century around Mount Elgon is more than 500 (Kitutu 2010; Nakileza et al. 2017). Although the number of PWDs has not always been brought out among landslide-affected victims, several studies have revealed that disasters double the impact when it comes to PWDs (Cerimovic & Rall 2021; International Federation of Red Cross and Red Crescent Societies (IFRC) 2021; UN 2018; UNDP India 2012; UNISDR 2014). This has made many unknown lives of PWDs perish without documentation because these are largely ignored by society (IFRC 2021; UN 2018).

Disaster impact on PWDs worsens in areas with rugged terrain where accessing appropriate transport, logistics, and evacuation warnings become harder (WID 2018). In the face of inaccessibility and landslide hazards, PWDs are not able to access personal support networks (including family, friends, or paid caregivers) for well-being and independence due to difficulties in moving, seeing, and hearing, among others (Bagonza 2014; WID 2018). Altitude, stream power for the flowing water, slope angle, hillslope position, curvature, and slope aspect are the major factors underpinning landslide susceptibility (Bamutaze 2019; Kozak, Ostapowicz & Bytnerowicz 2013; Nohani et al. 2019).

The National Inclusive Planning Guideline for Uganda caters for only PWDs affected by constructions and refugees for resettlement and less interest is put on those who are affected by disasters (NPA 2017). Lord et al. (2016) states that PWDs suffer consequences of disasters uniquely depending on the disability. There is a need to avail information and knowledge regarding the susceptibility of PWDs to landslide risk in Bushika sub-county, the epicentre of landslide occurrence to advocate for the prioritisation of PWDs in resettlement programmes for landslide victims launched by the government (IFRC 2021; Wambede & Kolyangha 2019). Our objective was to use Geographical Information Systems (GIS) tool and map the susceptibility of PWDs to landslide hazards and investigate the perceived disparity of landslide effect with the disability category.

## Methodology

### Description of the study area

**FIGURE 1:**
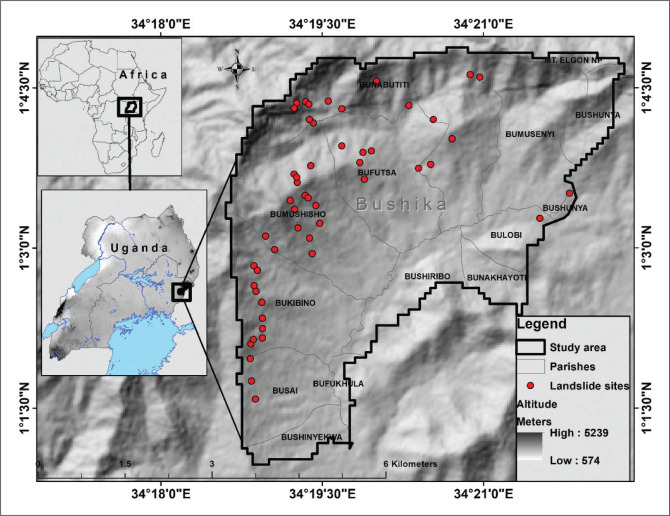
Showing the mapped landslide sites.

The study was carried out in Bushika sub-county, which lies in the northern parts of Bududa district in Mount Elgon catchment of Eastern Uganda as shown in Figure 1. It lies between Longitudes 34°18.5’ 0’’ E to 34°22.5’0’’ E and Latitudes 1°1’0’’ N to 1°5’0’’ N. It has an approximated area of 17.5 km^2^. Bushika sub-county has the highest disability prevalence of 22.8% in Bududa district (UBOS 2019). The area has an altitude ranging between 1400 m a.s.l. in the South to 2163 m a.s.l. in the West. Bushika is one of the ridges in Bududa alongside Bulucheke, Bukighai, Bukalasi, Nakatsi among many others. Bushika is dominantly covered with Luvisols. These soils are yellowish-brown sand clay loams with the Elgon Volcanic and basement complex granite rock. Luvisols have medium to high productivity. The climate is determined by the irregular moist south-westerly and dry north-easterly air streams.

**FIGURE 2:**
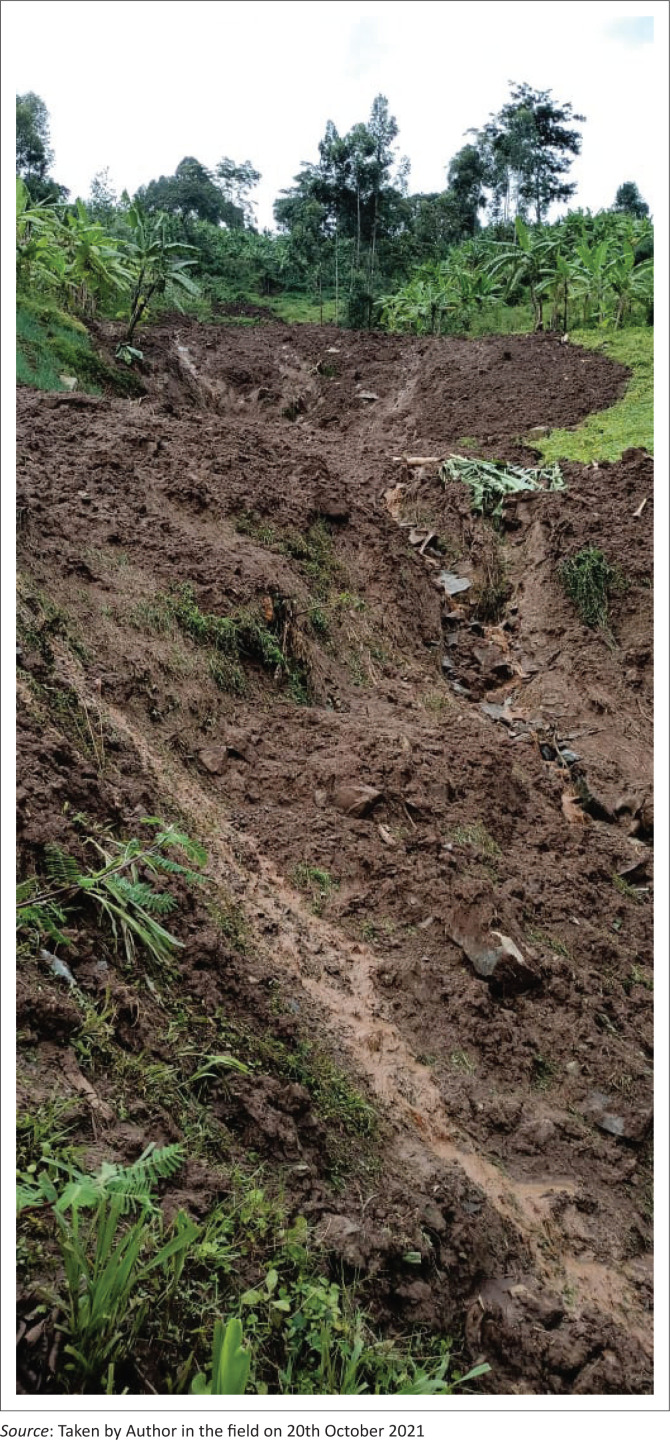
Landslide that occured following a heavy downpour in the night 14, Oct 2021, affecting 214 (33 households) and displacing 65 people in Nakhatore village, Bududa district.

**FIGURE 3:**
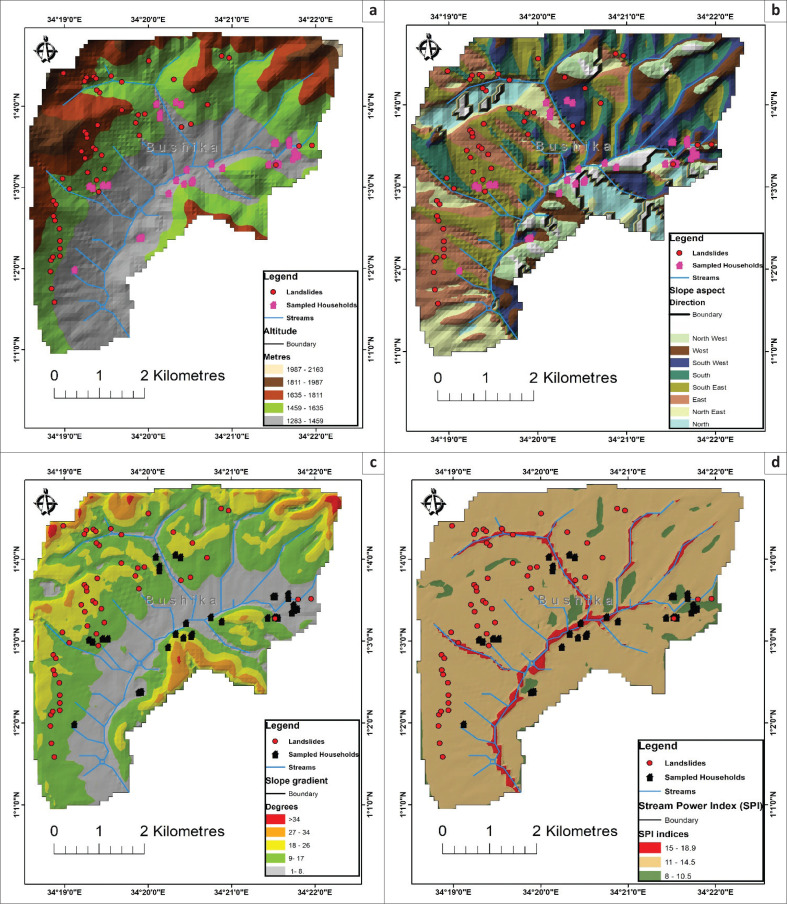
Landslide risk mapping for person with disability households using terrain factors of (a) Altitude; (b) Slope aspect; (c) Slope gradient; (d) Stream Power Index.

The area experiences a bimodal rainfall pattern. The wettest period of the year is from March to October, while the dry season occurs from November to February with a short dry period around July with rainfall ranging from 1500 mm/yr to 2000 mm/yr (Kitutu 2010). The main land-use type is agriculture (farming and small-scale grazing) and forest (national park). The dominant crops are perennial (banana and coffee), which are intercropped with beans and maize (Nakileza et al. 2017). Bushika sub-county is estimated to have a total number of 31 530 people with 17.54 km^2^ with 4.5 annual population increase. and a population density of 1773 persons per km^2^ based on Bushika sub-county population projection 2020 chart. The estimated number of PWDs is 4729 (UBOS 2019). The total population in Bududa district is approximately 210 173 people, with a 15.2% disability prevalence. The National Analytical Report by UBOS (2019) on PWDs, recorded a higher fertility rate for PWDs of 6.3 which is 0.5 more than 5.8, the fertility rate for non-PWDs in Uganda. Bushika sub-county has a high occurence of landslide hazards in Mount Elgon area as shown in Figure 3 and Figure 5.

### Research design

A cross-sectional research design with in-depth interviews was adopted. A proportionate sample of 55 households having one or more PWDs with 18 years of age was used for the study. The estimated households with PWDs above 18 years in Bududa were 10 317 and 1461 for the Bushika sub-county (UBOS 2019). Using the overall sample size of Bududa district (385), a sample for Bushika was scientifically derived using Cochran’s 1977 formula of proportionate sample size determination. Because the majority of the PWDs live uphill and are isolated, snowball sampling was employed to easily reach out to the households of PWDs. Global Positioning System (GPS) receivers were used to capture the coordinates for the households and landslide scar sites during data collection. A unique identifier was marked on the used questionnaires (García et al. 2019). The questionnaire was designed with a section to obtain landslide effect to the disability categories based on respondent’s perception. A five-point Likert scale ranging from 1 (less affected) to 5 (most affected) was used to assess the perceived landslide hazard effect. The respondents’ experience and perceived knowledge is appropriate for participatory resilience enhancement especially in areas of high disaster risk (Ahmad, Ahmad, Reza Arman 2020; Bodrud-Doza et al. 2020; Khan & Baba 2017). Key informant interviews were also used to collect data from the following personnel: 1) Bududa District Environment Officer, 2) the District Community Development Officer, 3) District Disaster Focal Person/Deputy Chief Administrative Officer, 4) the female District Councilor for PWDs 5) the male District Councilor for PWDs 6) Male Councilor PWDs of Nangako Town Council, Bududa and 7) the Chairperson Bududa District Union of PWDs. Also, two focus group discussions comprising male and female participants were conducted.

### Data analysis

#### Landslide terrain parameters

Terrain parameters selected to determine landslide risk were Hillslope, altitude, stream power, slope angle, curvature, and slope aspect. The choice of these parameters was based on existing literature of studies by Nakileza and Nedala (2020) and Bamutaze (2019) that underpin them as most influential in landslide occurrence for Elgon areas. The mid altitudes of 1500 m – 1800 m are classified to be more susceptible to landslides. Besides, sites with higher stream power indices (SPI) tend to be more erosive, and therefore have higher risks of gulley formation and consequently detonating downslope debris movements (Nseka, Kakembo & Mugagga 2019). Also, landslide risk increases with slope gradient and then reduces at steeper slopes (Bamutaze 2019). Middle slopes are generally susceptible to landslides (Nugraha et al., 2015; Hosseini et al. 2011). The local slope aspect influences weathering process and landslide occurrences on the hillslopes (Masoumi, Jamali & Khabazi 2014; Nohani et al. 2019). Also, Planula curved hillslopes are highly susceptible to large landslides in Mount Elgon areas (Nakileza & Nedala 2020).

#### Raster processing using GIS

The hillslope position was recorded while in the field based on the seen hillslope configuration. A 30 m SRTM Digital Elevation Model (DEM) for Uganda was downloaded from Regional Center for Mapping of Resources for Development (RCMRD) (https://opendata.rcmrd.org/). Digital Elevation Model filling to remove small imperfections such as sinks and peaks was executed to improve accuracy during the delineation of basins and streams (Planchon & Darboux 2001). The DEM coordinate system was transformed from GCS WGS 1984 to WGS 1984 UTM Zone 36N, better for GIS analysis. After DEM filling, extraction by mask using the delineated boundary of Bushika sub-catchment was executed. The extracted DEM for the sub-catchment was then used for spatial terrain analysis. In ArcGIS, altitude, curvature, aspect, and slope gradient (0°) were automatically processed using spatial analyst tools in ArcMap following the steps advanced by Burrough and McDonell (1998). Stream Power Index (SPI) was derived using the terrain hydrologic tools in System Automated Geoscientific Analyses (SAGA) GIS using a formula denoted as:

SPI=SCA×tan(Slope) [Eqn 1]

where SCA, is the specific catchment area.

#### Quantitative and qualitative data analysis

The perceived variation of landslide effect with different disability categories was obtained with quantitative approach and later analysed using a one-sample T-test in SPSS version 23. The one sample T-test is regarded as robust and versatile in analysing scale data (Vieira 2016). Content analysis was used on the captured story data during the survey. This qualitative data was crucial in explaining the results from the quantitative queries.

### Ethical considerations

The study was approved by the Department of Geography, Geo-informatics and Climatic Sciences and the Graduate Research Committee at Makerere University. Permission to carry out data collection was given by the Chief Administrative Office (CAO) of Bududa District and the Chairman of the District Disaster management committee (DDMC).

## Results

### Susceptibility of persons with disabilities to landslide hazards

The results showed that PWDs were highly scattered with a majority (69%) living in altitudes of 1317 m – 1700 m above sea level. With slope aspect, most of PWDs (38%) lived on south-eastern and south-western facing hillslopes (19%). The number of PWDs who lived on the hillslopes facing in the south and north-western was (32%). Each of the hillslopes with aspects north, north-east, and east had 9% PWDs. Only 3% were living on hillslopes facing in the western direction. The dominant number of PWDs (56%) were living in areas of slope gradient ranging from 9° to 26° with only 44% living in sites with 1° – 8°. Most of the PWDs (69%) lived in areas with high stream erosive power (SPI) ranging from 12 to 19. Only 31% were living in areas with medium power of flowing streams in the area. The majority of PWDs (46%) were living in middle slope areas followed by those who were located at the foothills (41%) as shown in Table 1. Only 12% had their homesteads located at hill summits or flat areas of the slope as shown in Figure 3. It was observed that most of the PWDs settled in areas far from the towns and trading centres in the area such as Bushika and Nangako trading centres.

**TABLE 1:** Landslide risk of persons with disabilities’ (PWDs) households due to slope positions.

Disability	Number of PWDs %			Total (%)
	Foot	Middle	Summit/top	
Blindness	3.1	3.1	3.1	9
Deaf	0	6.3	0	6
Physical impairment	13	34	6.3	53
Deaf blind	3	0	0	4
Multiple disability	22	3	3	28

**Total**	**41.1**	**46.4**	**12.4**	**100**

**FIGURE 4:**
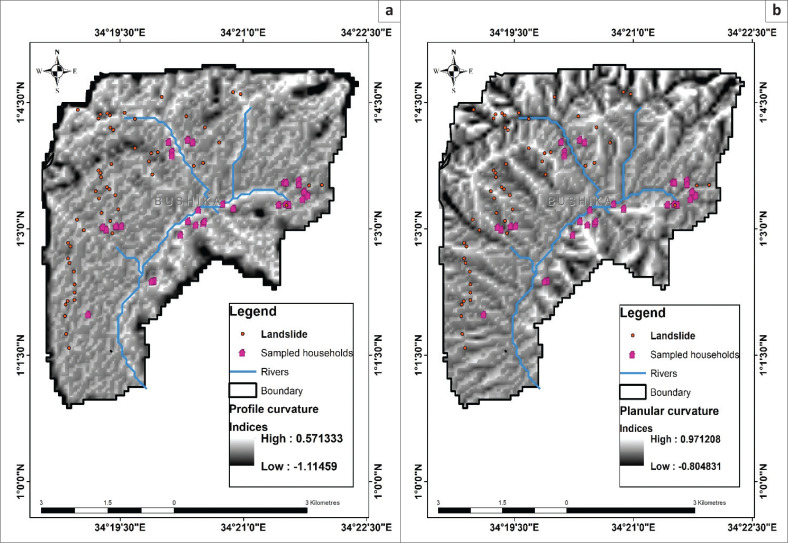
Landslide susceptibility of households for persons with disabilities due to slope curvature of (a) Profile curvature and (b) Planula curvature.

The results of slope curvature concerning the dwellings of PWDs are presented in Table 2 and further illustrated in Figure 4. Concerning the Planula curvature as an attribute of slope curvature, the majority of PWDs (84%) lived in areas with curvature indices of –0.804 to –0.129, with a few who lived in areas with 0.129–0.97. Concerning the locations of the PWDs and profile curvature, a majority (53%) were living in sites with curvature indices ranging from 0.055 to 0.57. The other PWDs (47%) were living in areas with –0.228 to 0.055 curvature indices.

**TABLE 2:** Summary of the perceived landslide effect on disability categories.

Disability category	A measure of the difference in a landslide effect on various disability categories								Sum of score	Landslide effect
	*t*	*df*	*p*	Mean difference	95% confidence interval		Standard deviation	Standard error mean		
					Lower	Upper		
Blind	58.4	31	0.000	4.71875	4.5541	4.8834	0.45680	0.08075	151	Most
Deaf	34.5	31	0.000	3.90625	3.6753	4.1372	0.64053	0.11323	125	High
Physical	24.2	31	0.000	3.40625	3.1187	3.6938	0.79755	0.14099	109	Affected
Mental	18.5	31	0.000	2.09375	1.8628	2.3247	0.64053	0.11323	67	Moderate
Multiple	17.3	31	0.000	3.37500	2.9785	3.7715	1.09985	0.19443	108	Affected
Little people	19.2	31	0.000	2.18750	1.9551	2.4199	0.64446	0.11392	72	Moderate
Epilepsy	12.5	31	0.000	2.25000	1.8837	2.6163	1.01600	0.17961	52	Moderate
Deaf-blind	34.8	31	0.000	4.62500	4.3541	4.8959	0.75134	0.13282	148	Most

**FIGURE 5:**
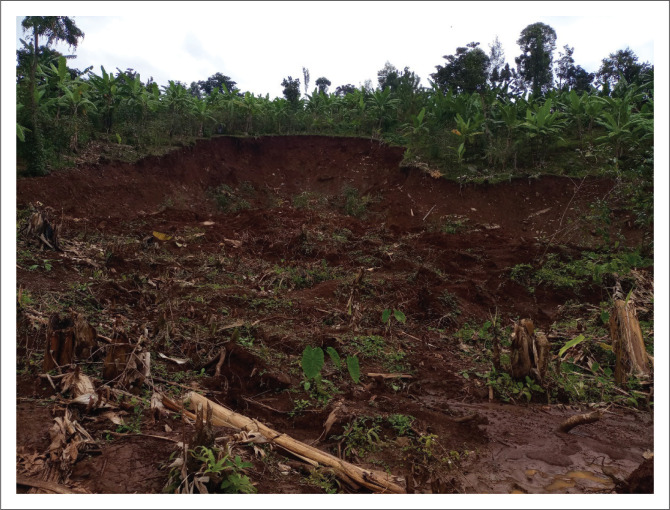
Landlside that occurred at 12:00 noon and then re-occurred at 15:00 covering villages of Mayila, Nabutatsi and Kikonyelo in Bududa district where more than seven households and a church were demolished including those of persons with disabilities as reported by the nearby locals and host families.

During a focus group discussion, PWDs also reported that they live in areas highly susceptible to landslides:

‘… In this area, the frequence of landlside occurrence has increased, landslides used to occur after decades but now, we can experience two landlide events in one month‥we still expect landlsides to occur…‥up there on this hillslope, there are still linning cracks ….’

### Perceived landslide effect on disability categories

The results of the perceived degree of landslide effect on disability are presented in Table 3. One sample T-test showed that blind (151) and deaf-blind (148) were perceived as most affected by landslides with *t*(31) = 58.42, mean = 4.7, *p* < 0.0001, and t(31) = 34.8, mean 4.6, *p* < 0.0001. Besides, the deaf were highly affected by landslides with *t*(31) = 34.4, mean = 3.9, *p* < 0.0001. Persons with disabilities especially deaf-blind persons were reported to be last during recovery and rescue in case of landslides. A key informant interview with the District Community Development Officer revealed that:

‘… In case of a landslide disaster, those PWDs are considered last, priority goes to the children, breastfeeding mothers, elderly and then PWDs….’

**TABLE 3:** Respondents’ characteristics.

ID	Altitude (m)	Assistive Device	Duration of stay (years)	House ownership	Hillslope position	Difficulty in
1	1461	No	All my life	Own	Middle	Walking
2	1423	No	All my life	Own	Foots lope	Communicating
3	1458	No	All my life	Own	Foots lope	Multiple
4	1457	No	All my life	Own	Foots lope	Multiple
5	1436	No	All my life	Own	Foots lope	Multiple
6	1448	No	All my life	Own	Foot slope	Walking
7	1481	No	All my life	Own	Middle	Multiple
8	1434	No	All my life	Own	Foot slope	Multiple
9	1340	No	All my life	Own	Foot slope	Seeing
10	1503	yes	All my life	Own	Middle	Walking
11	1378	No	All my life	Own	Top	Walking
12	1383	yes	All my life	Own	Top	Seeing
13	1393	No	Past few years	Own	Foot slope	walking
14	1479	No	All my life	Own	Middle	Walking
15	1490	yes	All my life	Own	Middle	Hearing
16	1496	No	All my life	Own	Middle	Walking
17	1566	No	All my life	Own	Middle	Walking
18	1700	yes	All my life	Own	Top	Walking
19	1317	yes	Past few years	Others	Middle	Seeing
20	1632	No	Past few years	Own	Middle	Walking
21	1547	yes	All my life	Own	Middle	Walking
22	1383	No	All my life	Own	Foot slope	Multiple
23	1384	yes	Past few years	Own	Foot slope	Walking
24	1393	No	All my life	Own	Middle	Walking
25	1538	No	All my life	Own	Middle	Walking
26	1372	yes	All my life	Own	Foot slope	Hearing
27	1378	No	All my life	Own	Top slope	Multiple
28	1403	yes	All my life	Own	Middle	Walking
29	1453	No	Past few years	Own	Foot slope	Walking
30	1450	No	Past few years	Own	Foot slope	Multiple
31	1457	No	All my life	Own	Foot slope	Multiple
32	1465	yes	All my life	Own	Foot slope	Walking

Results depicting blind and deaf-blind as most affected by landslide hazards were due to the inability to self-evacuate to safer places especially during landslide occurrence where able-bodied persons and caretakers easily run for their lives.

The Chairman of Bududa District Union of PWDs during an interview postulated that:

‘… The most affected people with disability when landslides occur are the blind, deaf-blind, and physically impaired but unable to move. This is because persons with difficulty in seeing (the Blind) can’t see cracks and the deaf can’t hear early warning messages, compared to those with physical disabilities. In times of landslide emergencies, caretakers and guides also run away, living the Blind and Deaf exposed to the risk of landslides….’

The little people (72), those with mental (67) and epileptic (51) were moderately affected by the landslide hazards in Bushika with t(31) = 19.2, mean = 2.2, *p* < 0.0001, t(31) = 18.5, mean = 2, *p* < 0.001 and t(31) = 12.5, mean = 2.2, *p* < 0.0001. This was on account of the fact that they are able to hear and sense the direction of the landslide and call for intervention while advancing to safer places independently or with minimal support. Furthermore, a female District Councilor for PWDs in Bududa revealed that:

‘… During the occurrence of landslides, PWDs especially the Blind and deaf are hit highest because the household members usually scatter to different directions leaving them without assistance, for example in the last landslide occurrence, a deaf member lost two kids in Bushika Sub County and a blind girl was raped in Bulucheke in the aftermath of a landslide as she was seeking guidance to a safe place….’

## Discussion

The higher numbers of PWDs were living in high-risk areas with altitude, slope gradient, slope aspect, and SPI ranging from 1400 m.a.s.l to 1700 m.a.s.l especially midslopes, 9° to 26°, S-E to S-W, and 12–19 respectively. The dwelling locations for majority of PWDs were recorded at slope angle and altitude ranging from 11° to 31° and 1500 m.a.s.l to 1900 m.a.s.l., respectively. These ranges are in line with those Bamutaze (2019) revealed as sites with the highest prevalence of landslides during his study on morphometric conditions underpinning the spatial and temporal dynamics of landslide hazards on the volcanic Mount Elgon, Eastern Uganda. With aspect, PWDs lived on slopes facing S-E and S-W which are highly susceptible to landslides as revealed by Nakileza and Nedala (2020). Concerning susceptibility to erosion and gulley formation at their places of living, the bulk of the PWDs lived in locations with SPI beyond > 10 with higher erosive power of the flowing water in the streams, thus higher chances of gully formation and consequently becoming susceptible to landslides. The bulk of PWDs lived in sites with planar curvature indices of –0.804 to –0.129 which were noted by Nakileza and Nedala (2020) as highly susceptible to landslides in their study of topographic influence on landslides characteristics and implication for risk management in Mount Elgon. Sites with negative planar curvature indices cause materials to converge, thus escalding water running consequently triggering debris flow downhill. Such sites are highly risky for settlement by PWDs. Although, Neema et al. (2018) revealed that low-lying locations and trading centres in Bududa are safer with low landslide risks, some PWDs have homes in middle and foot slope places because that is the land they can afford. The study results are in line with a baseline study by Uganda National Action on Physical Disability (UNAPD) (2016) where PWDs indicated that they stay uphill with high-risk locations of landslides in Mount Elgon area. In addition, during his study on gender and vulnerability to disasters, Bagonza (2014) also observed that many people construct houses and settle on high-risk hills in Uganda. According to Neema et al. (2018), people continue to settle in the high-risk areas, because people seem to be uncertain and have mistrust in the implementation of the resettlement programmes, especially on issues of compensation for land lost. The first-ever UN global survey of persons living with disabilities UNISDR (2014) revealed that PWDs are rarely consulted about their needs in potential disaster situations and thus excluded from the planning and decision-making of such processes. The higher number of the peasantry, poverty levels, and failure to afford safer places also account for the consistent settlement of PWDs in high-risk areas. The discrimination by society precisely accounts for settlement in isolated areas. However, these hard-to-reach areas make it difficult to receive the services, information, resources including early warnings and recovery as echoed by the PWDs and their leaders.

Findings on the variation of landslide effect where the blind and Deaf were perceived as most affected by the landslide hazards corroborate those of Madanian et al. (2018) and Lord et al. (2016). They have also found out that people with visual impairment, and particularly the older adults are most affected in times of crisis and emergency especially earthquakes and landslides that vibrate and shake land thus causing more tension. Furthermore, a report by ENPCM (2017), also found similar results that people with visual impairment have a greater susceptibility to a variety of hazards and potential threats in daily life. This is explained by a higher risk of stress reactions and social isolation in case of crisis. The results on the disproportionate damages caused by landslides are in line with the findings by Twigg et al. (2018). The difference in the impact of landslides on the different PWDs categories is attributed to the dissimilarity in the societal and formal discrimination, disregarding, and exploitation experienced by PWDs in disasters.

## Conclusion

Persons with disabilities live in risky areas and yet these are highly affected during a landslide crisis. The blind and Deaf-blind were perceived as most affected by landslides hazards. Therefore, blind and deaf-blind persons should be prioritised for relocation to safer areas within their ancestral district due to cultural attachment and fertile soils. In addition, landslide prone areas such as Bududa, disaster programs and committees at village should include PWDs to further strengthen awareness, inclusion, access to early warning information, disaster survival tips during hence strengthening their resilience to these recurrent landslides.
